# Identifying Fatigue Indicators Using Gait Variability Measures: A Longitudinal Study on Elderly Brisk Walking

**DOI:** 10.3390/s20236983

**Published:** 2020-12-07

**Authors:** Guoxin Zhang, Ivy Kwan-Kei Wong, Tony Lin-Wei Chen, Tommy Tung-Ho Hong, Duo Wai-Chi Wong, Yinghu Peng, Fei Yan, Yan Wang, Qitao Tan, Ming Zhang

**Affiliations:** 1 Department of Biomedical Engineering, The Hong Kong Polytechnic University, Hong Kong 999077, China; guo-xin.zhang@connect.polyu.hk (G.Z.); ivy-kk.wong@polyu.edu.hk (I.K.-K.W.); tony.l.chen@connect.polyu.hk (T.L.-W.C.); tommyth.hong@connect.polyu.hk (T.T.-H.H.); duo.wong@polyu.edu.hk (D.W.-C.W.); 18041923r@connect.polyu.hk (Y.P.); bme-fei.yan@polyu.edu.hk (F.Y.); yawang@polyu.edu.hk (Y.W.); matthew.tan@connect.polyu.hk (Q.T.); 2 Shenzhen Research Institute, The Hong Kong Polytechnic University, Shenzhen 518057, China

**Keywords:** fatigue, brisk walking, kinematics, gait, inertial measurement unit

## Abstract

Real-time detection of fatigue in the elderly during physical exercises can help identify the stability and thus falling risks which are commonly achieved by the investigation of kinematic parameters. In this study, we aimed to identify the change in gait variability parameters from inertial measurement units (IMU) during a course of 60 min brisk walking which could lay the foundation for the development of fatigue-detecting wearable sensors. Eighteen elderly people were invited to participate in the brisk walking trials for 60 min with a single IMU attached to the posterior heel region of the dominant side. Nine sets of signals, including the accelerations, angular velocities, and rotation angles of the heel in three anatomical axes, were measured and extracted at the three walking times (baseline, 30th min, and 60th min) of the trial for analysis. Sixteen of eighteen participants reported fatigue after walking, and there were significant differences in the median acceleration (*p* = 0.001), variability of angular velocity (*p* = 0.025), and range of angle rotation (*p* = 0.0011), in the medial–lateral direction. In addition, there were also significant differences in the heel pronation angle (*p* = 0.005) and variability and energy consumption of the angles in the anterior–posterior axis (*p* = 0.028, *p* = 0.028), medial–lateral axis (*p* = 0.014, *p* = 0.014), and vertical axis (*p* = 0.002, *p* < 0.001). Our study demonstrated that a single IMU on the posterior heel of the dominant side can address the variability of kinematics parameters for elderly performing prolonged brisk walking and could serve as an indicator for walking instability, and thus fatigue.

## 1. Introduction

Physical exercise is imperative for the elderly to maintain and promote a healthy lifestyle [1]. At least two and a half hours of moderate-intensity aerobic physical activity per week has significant health effects on the health of the elderly [2,3]. In addition, regular physical activity can reduce the risk of falls by 30% for the poor-mobility elderly [4]. It is recommended to integrate physical activity, such as walking, into daily routines [5] since it is convenient [6]. The elderly are recommended to walk 5000 steps per day to minimize the falling risk [7] and even encouraged to walk 7000 to 10,000 steps per day for health benefits [8]. However, the decline in muscle strength, coordination, and motor perception as aging impairs older people’s balance control ability increases the fear in older adults of falling [9]. As a result, high nervousness caused by the fear of falling and decreased muscle strength and tolerance make the elderly more prone to fatigue, which is associated with falling [10], and falling poses a serious threat to the elderly [11]. Therefore, timely identification of fatigue can reduce the risk of falls and enable the elderly to exercise more for better health.

Previous studies have found some indicators related to walking fatigue for the elderly. Fatigue attenuated the range of motion and muscle strength of the ankle joint of the dominant leg [12], in addition to the variability of step width and the minimum foot clearance (MFC) that were regarded as risks of tripping [10]. Gait asymmetry and variability measures are indirect indexes to evaluate fatigue [13]. Some researchers attempted to verify neuromuscular fatigue of lower limb and trunk muscles using electromyography [14,15]. The decreased coactivation of the ankle joint in the first half of the swing phase was found to be correlated with muscle fatigue in walking [16].

The application of an inertial measurement unit (IMU) is becoming popular recently due to its superiority in portability, cost, and accessibility compared to traditional gait labs with motion capture systems and force platforms [17]. IMUs were used to indirectly classify the fatigue of lower extremity muscles [18], to detect age-related gait deviation [19], and to direct balance improvement programs [20].

While there is an abundance of research on the walking fatigue of the elderly, the measurement (including the baseline and fatigue state) was often standalone from the fatigue-inducing task (e.g., long-distance walking [13]). This may or may not reliably acquire the state of fatigue from participants. Protocols in existing studies may allow the participants to recover when they switch from the fatiguing tasks to the test for measurement.

In this light, we propose to collect the kinematic data throughout the whole fatigue-inducing tasks for analysis without a standalone testing trial for measurement. This approach could ensure that the participants will not recover halfway, and the fatigue-inducing effect could be continuous. In addition, the advantage of this study lies in its potential to predict instability or fatigue using a single sensor measuring the orientation, angular velocity, and linear acceleration of the posterior heel. The main purpose of this study is to study the influence of fatigue induced by brisk walking on gait parameters by using an IMU and identify fatigue indicators. It is hypothesized that lower-limb muscle fatigue will manifest the deterioration of stability and increase energy consumption. This study is not only helpful for the elderly’s fast walking exercise but also the development of wearable fatigue recognition equipment, the elderly’s fall prevention, and rehabilitation training.

This paper includes five parts. The Introduction section covers the research background and significance of fatigue indicators. The Materials and Methods section provides the information of participants, experimental protocol, outcome measures, and statistical analysis. The Results section highlights the changes in outcome measures across the time of long-term brisk walking as well as the results of the statistical analysis. Subsequently, we address the implications of our key findings and the limitations of our study in the Discussion section. Lastly, we conclude the feasibility of our system on fatigue detection with a cautious note on the relationship between the variability of kinematics and fatigue.

## 2. Materials and Methods

### 2.1. Participants

Eighteen older adults, nine males and nine females, were recruited from the university campus by posters and leaflets. The average age, height, and weight of the participants were 60.4 ± 7.9 years, 161.0 ± 7.6 cm, and 62.7 ± 11.7 kg, respectively. All these participants were independent walkers with a heel strike walking style. The exclusion criteria included persistent knee pain, osteoarthritis, unstable ankle, severe flat foot, severe hallux valgus, toe deformities, and other chronic diseases that might lead to safety issues. The participants shall also have no history of injuries in the past six months. All participants signed the informed consent, and this study was approved by the Human Subjects Ethics Sub-committee of The Hong Kong Polytechnic University (Reference Number: HSEARS20190919001).

### 2.2. Equipment and Experimental Procedures

All participants wore the same type of cushioned running shoes, ARHQ025-4 (Li-Ning Inc., Beijing, China), with self-selected sizes, from 36 to 43 (European size). The insole, upper, and outsole are made of Ethylene-vinyl acetate copolymer (EVA), synthetic, and rubber, respectively.

The closure type is lace-up. Before the running task, the safety precautions in using the treadmill (Unisen Inc., Tustin, CA, USA) and the general procedures of the experiment were explained to the participants. By increasing the treadmill speed by 0.5 km/h every 30 s [16], participants reached their self-preferred comfortable brisk walking speed. After 5 min of familiar walking on the treadmill, participants brisk walked for 1 h at this speed. The mean preferred comfortable fast walking speed was 3.9 ± 0.6 km/h. After the brisk walking trial, the participants were asked whether they experienced no fatigue or a mild or severe level of fatigue.

**Figure 1:**
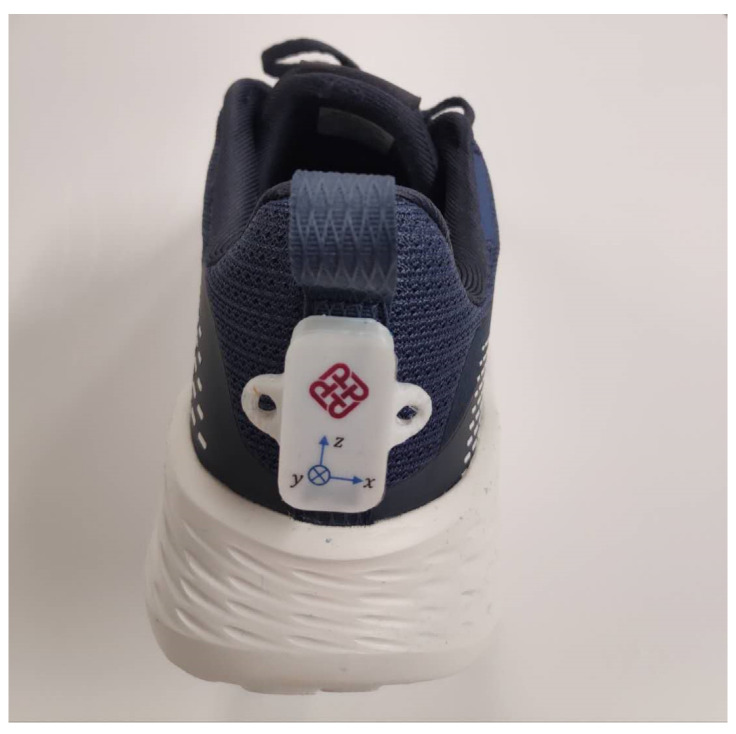
The placement and orientation of the inertial measurement unit (IMU) on the posterior heel region.

The acceleration, angular velocity, and angles of the posterior heel region of the dominant foot in the anterior–posterior axis, medial–lateral axis, and vertical axis were measured using a single IMU (BWT901BLE5.0, Wit motion Inc., Shenzhen, China). The hardware of the IMU utilized an MPU 9250 (InvenSense Inc., CA, USA). The software of the IMU integrated dynamics calculation and the Kalman filter algorithm and provided a mobile application that can collect data in real time. The IMU was attached on the middle posterior heel of the dominant foot as shown in Figure 1, sampling at 50Hz. The specifications of BWT901BLE5.0 are shown in Table 1 and the full-scale ranges of the accelerometer and gyroscope were ± 16 g and ± 2000 °/s. The x-axis, y-axis, and z-axis corresponded to the medial–lateral axis, anterior–posterior axis, and vertical axis, respectively, as shown in Figure 1. The dominant limb was determined by asking participants to kick a ball five meters away from the goal [21].

### 2.3. Data Processing

The average baseline orientation of the IMU was measured when the participants walked for the first 30 s of the walking trial. The calculated angles referred to the changes in orientation angle compared to the average baseline orientation.

We extracted the baseline (1st-min), 30th-min, and 60th-min data for analysis. The outcome measures included the acceleration, angular velocity, and angle in the anterior–posterior axis, medial–lateral axis, and vertical axis. Ten parameters were derived from these three time series’ primary data including the median absolute deviation (MAD), kurtosis, skewness, root mean square (RMS), variance, maximum absolute value, minimum absolute value, amplitude range, the median of absolute value, and the average of energy consumption (EC). In this study, signal power was regarded as EC, and the signal power of a real-valued signal x(t) is defined as follows [22]:

(1) $$P=\underset{T\to \infty }{\lim}\frac{1}{2T}\int_{-T}^{T}{\left|x\left(t\right)\right|}^{2}dt$$

The process of data processing is summarized as follows.

1. Extract the data of the baseline min, 30th min, and 60th min, and incomplete gait cycles are ignored.
2. Calculate these 10 parameters of each gait cycle in these three time periods, then calculate the average value for each parameter.
3. Normalize by dividing each participant’s baseline-min, 30th-min, and 60th-min data by their respective baseline-min data to get the proportion of change of each parameter relative to the baseline, thereby eliminating the difference in gait parameters between the experiments. The units of each parameter are 1 since each parameter uses a ratio.

Take the RMS of the 30th min as an example. The baseline min, the 30th min, and the 60th min consist of $n_{1},\;n_{30},\;n_{60}$ gait cycles, respectively. The calculation of the 30th min’s RMS is

(2) $${\mathrm{RMS}}_{30}=\frac{\mathrm{RMS}\_30}{\mathrm{RMS}\_1}=\frac{\frac{1}{n_{30}}\sum_{i=1}^{n_{30}}\mathrm{RMS}\_30_{i}}{\frac{1}{n_{1}}\sum_{i=1}^{n_{1}}\mathrm{RMS}\_1_{i}}$$

### 2.4. Statistical Analysis

Statistical analysis was used to determine whether there were statistically significant differences in the outcome measures (detailed in Section 2.3) over the course of the brisk walking trial at the baseline, 30-min, and 60-min time points.

The data were not normally distributed as assessed by the Shapiro–Wilk test (*p* > 0.5). Therefore, the nonparametric test (Friedman test) was used. The significance level (α) was set at *p* = 0.05. If the findings of the Friedman test were statistically significant, a post hoc pairwise comparison, the Wilcoxon signed-rank test, was conducted with Bonferroni correction at *p* < 0.017.

## 3. Results

After the brisk walking trial, 12 out of the 18 participants reported severe fatigue and 4 participants reported a mild level of fatigue, while the remaining two participants reported non-fatigue. The outcome measures from the IMU after data processing are shown in Table 2. Twenty-five outcome variables demonstrated a statistically significant difference among the walking time conditions in the Friedman test. A post hoc pairwise comparison was conducted on the outcome variables with significance, as shown in Table 3.

### 3.1. Influence of Walking Time (Fatigue) on Posterior Heel Acceleration

There were eight significant differences in statistical features in the three axes’ accelerations, as shown in Table 2, including the range (*p* < 0.03) and median (*p* < 0.002) of the x-axis acceleration, the RMS (*p* < 0.03), variance (*p* < 0.03), minimum (*p* < 0.002), and EC (*p* < 0.03) of the y-axis acceleration, and the skewness (*p* < 0.03) and median (*p* < 0.03) of the z-axis acceleration.

**Figure 2:**
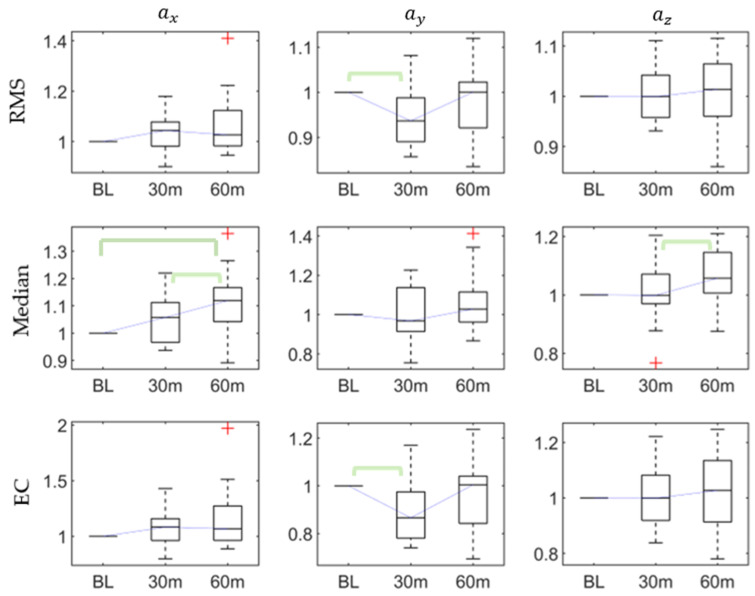
The root mean square (RMS), median, and energy consumption (EC) of accelerations at baseline, 30th min, and 60th min. Bracket denotes statistical significance (*p* < 0.05) in the post hoc analysis with Bonferroni correction.

The post hoc pairwise comparison test (Table 3) revealed that, for $a_{x}$, there were significant differences in the range between the baseline and 60th min, the median between the baseline and 60th min, and the 30th and 60 min, and the RMS between the baseline and 30 min. For $a_{y}$ there were significant differences in the variance between the baseline and 30th min, the minimum between the baseline and 60th min, and 30th and 60th mins, and the EC between the baseline and 30th min. For $a_{z}$, there were significant differences in the skewness between the baseline and 60th min and the median between the 30th and 60th mins. Figure 2 shows that the RMS and EC of $a_{y}$ decreased after the first 30-min walking task, while the median of $a_{x}$ increased after the continued 30-min task (60-min).

### 3.2. Influence of Walking Time (Fatigue) on Posterior Heel Angular Velocity

**Figure 3:**
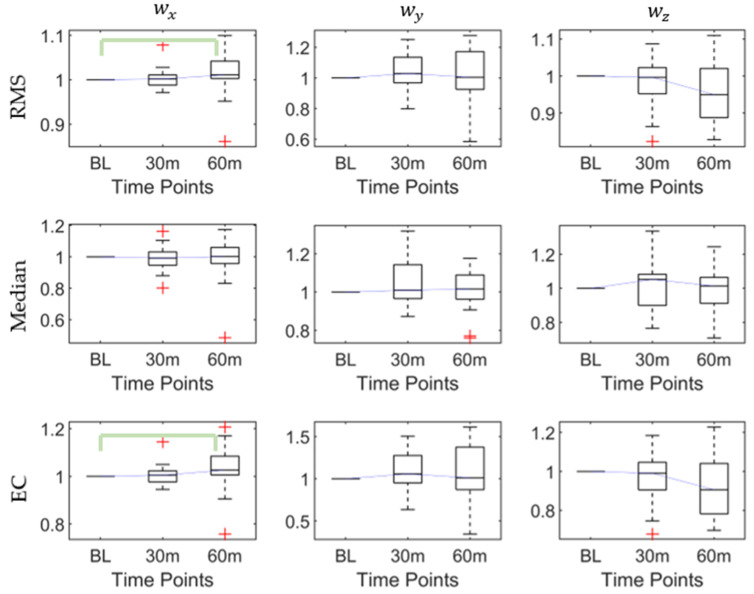
The root mean square (RMS), median, and energy consumption (EC) of angular velocities at baseline, 30th min, and 60th min. Bracket denotes statistical significance (*p* < 0.05) in the post hoc analysis with Bonferroni correction.

As Table 2 shows, there were significant differences in the RMS (*p* < 0.03), maximum (*p* < 0.03), and EC (*p* < 0.02) of the *x*-axis angular velocity, while there was no significant difference in the *y*-axis and z-axis angular velocities. The post hoc pairwise comparison test (Table 3) proved that the significant differences in the RMS, maximum, and EC exist at the baseline and 60th min, which all increased after 60 min. The post hoc results of the RMS, median, and EC characteristics of the three axes’ angular velocities are shown in Figure 3.

### 3.3. Influence of Walking Time (Fatigue) on Posterior Heel Orientation (Rotation Angle)

There were significant differences in the RMS (*p* < 0.03), maximum (*p* < 0.002), range (*p* < 0.002), and EC (*p* < 0.03) of the x-axis angular; the RMS (*p* < 0.02) and EC (*p* < 0.02) of the y-axis angular; and in the kurtosis (*p* < 0.02), skewness (*p* < 0.05), RMS (*p* < 0.003), maximum (*p* < 0.03), minimum (*p* < 0.006), range (*p* < 0. 006), median (*p* < 0. 006), and EC (*p* < 0.001) of the z-axis angular, as shown in Table 2.

**Figure 4:**
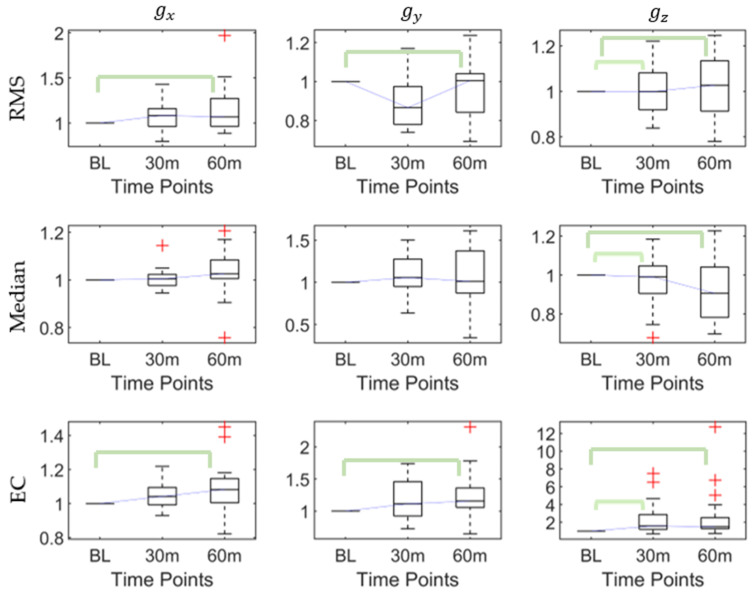
The root mean square (RMS), median, and energy consumption (EC) of angles at baseline, 30th min, and 60th min. Bracket denotes statistical significance (*p* < 0.05) in the post hoc analysis with Bonferroni correction.

The post hoc pairwise comparison test (Table 3) demonstrated that for $g_{x}$, there were significant differences in the RMS between the baseline and 60th min, the maximum between the baseline and 30th min, and the baseline and 60th min, and the range and EC between the baseline and 60th min; for $g_{y}$, there were significant differences in the RMS and EC between the baseline and 60th min; and for $g_{z}$, there were significant differences in the kurtosis and skewness between the baseline and 60th min and the RMS, maximum, minimum, range, median, and EC between the baseline and 30th min, and the baseline and 60th min. Figure 4 shows that the RMS, median, and EC of $g_{z}$ increased after the first 30-min walking session. After the continued 30-min task (60-min), the RMS and EC of $g_{x}$, the RMS and EC of $g_{y}$, and the RMS, median, and EC of $g_{z}$ all increased.

## 4. Discussion

This study explored the changes in kinematics and some derived kinematic variables after 60 min of brisk walking using an IMU. Our result showed that long-distance brisk walking may induce fatigue since most of the participants (16/18) responded that their subjective fatigue felt significantly increased, which imposed an apparent effect on the kinematic parameters of the individuals’ posterior heel of the dominant foot. After long-distance brisk walking, the participants showed a significant increase in (1) the median of acceleration in the medial–lateral axis ($a_{x}$), (2) maximum, variability, and energy consumption of angular velocity in the medial–lateral axis ($w_{x}$), (3) maximum, range, variability, and energy consumption of angle in the medial–lateral axis ($g_{x}$), (4) variability of angle in the anterior–posterior axis ($g_{y}$), and (5) median, maximum, range, variability, and energy consumption of angle in the vertical axis ($g_{z}$).

The increment in the medial–lateral acceleration indicates greater vibration of the dominant lower limb in the medial–lateral axis. This signal was likely manifested by a muscle fatigue condition since the acceleration signal reflects the mechanical activity of the muscle [23], which was advocated by previous studies [24]. The greater variation in medial–lateral acceleration could also be a sign of instability, imbalance, and falling risks [10,25,26]. Variation in step width could be related to age and falling [27] and distinguished between young and old adults [28,29,30] or between fallers and non-fallers [31,32].

The maximum angular velocity in the medial–lateral axis tended to increase after long brisk walking, and we assumed that this is a strategy to shorten the time of dominant foot swing and increases the double support time. This assumption was consistent with one previous study that suggested, in order to adjust the microbalance before the swing phase of the dominant limb becomes more unstable after fatigue, a longer double support time and shorter support time are required [16]. The increase in the variability of medial–lateral angular velocity demonstrated that the elderly were less stable after prolong brisk walking (or fatigue), which is consistent with previous studies [13,33]. A similar finding that elderly participants increased the median absolute deviation (MAD) of the medial–lateral ankle angular velocity after long-distance walking was found in [13]. In addition, the energy consumption increase indicates that they need more energy to maintain stability, which is consistent with the result of increased angular velocity variability.

After prolonged brisk walking, the maximum rotation angle in the medial–lateral axis became larger and could result in a larger rotation range. This phenomenon may represent a longer double support time, agreeing with the results of previous studies [10,16,18,34,35]. The rotation angle in the vertical direction also increased, and this proved that the instability induced by long-distance walking, or fatigue, may induce heel pronation. The altered lower limb alignment may increase the risk of overuse injuries [36]. This could be the reason why anti-pronation shoes were developed for pronated feet runners [37].

Furthermore, both variation and energy consumption of rotation angles increased in the three axes of anterior–posterior, medial–lateral, and vertical directions after long-distance brisk walking. This result is similar to a previous study that found, when the muscles adjacent to the joint are fatigued, there could be a decrease in the synergy between the neuromuscular feedback of the joints and the proprioception of the joints [16,18,38,39], resulting in a decrease in human balance control [40], deterioration in gait stability, and increased variability, which needs more energy to consume [16].

There were some limitations to this study. Due to the ethical considerations, elderly participants recruited in this study were generally more physically active and fit, to minimize the falling risks by this challenging prolonged brisk walking task. The physical fitness of the elderly people could be the confounding or effect modifier to the identifiers. In the real case, some elderly people could be reluctant to exercise and could be less physically tolerable. Sampling elderly people with different levels of physical fitness or risk of falls may improve the external validity of our findings, which could be conducted using postural stability tests [41,42]. On the other hand, we assumed that “fatigue” was induced by the prolonged brisk walking task as supported by the subjective feedback of the participant. However, in real practice, “fatigue” was difficult to evaluate and could be categorized into mental, neuromuscular, and metabolic fatigue [43]. We heavily relied on the presupposition of the indirect relationship between fatigue and instability (variation in parameters) supported by some literature [44,45]. It would be interesting to evaluate the sensitivity and specificity of a fatigue-predicting algorithm [46], in addition to the use of a receiver-operating chart (ROC), for optimization [47]. However, extensive effort is required to develop a validated instrument for precise fatigue measurement that warrants future study. Furthermore, future studies may apply electromyography [14], a musculoskeletal model [48], or near-infrared spectroscopy [49] to address and correlate different classes and intensities of fatigue. In addition, the integration of a multi-unit synchronized system could further facilitate activity monitoring [50], as well as reducing the sensor node complexity [51].

While the IMU system in this research aims to simplify and minimize the number of IMUs for fatigue identification, it should be noted that a number of research works have endeavored to investigate the influence of fatigue on variability, stability, and gait asymmetry [13]. Incorporation of these features may help improve the accuracy of fatigue prediction. A biofeedback system [52] could also be developed to let the elderly be aware of their fatigue or instability conditions and minimize falling risks.

## 5. Conclusions

This study utilized a single IMU to measure the linear acceleration, angular velocity, and rotation at the posterior heel during a 60-min brisk walking trial among elderly participants. The prolonged brisk walking induced a significant change in the signal parameters in the medial–lateral directions, including the linear acceleration, angular velocity, and heel rotation range. In addition, higher variability and energy consumption were also found on the angular velocity in the medial–lateral direction, as well as on the heel rotation angles in all directions.

Our study demonstrates that the brisk walking trial induced fatigue in the elderly participants as demonstrated by the subjective feedback. It should be noted that fatigue is a complex symptom, being the result of the complex interaction of physiological systems and the brain [53]. Since the brisk walking trial also induced changes in our measured signal parameters, we can conclude that our IMU-based system can identify fatigue status based on signal parameters. The findings in this study will pave the way towards developing fatigue-detecting smart shoes for elderly walking exercises to minimize the risk of falls.

## Figures and Tables

**Table 1:** The specifications of BWT901BLE5.0.

Parameters	Range	Stability	Transmission Frequency
Acceleration	±16 g	0.01 g	50 Hz
Angle	X/Z: ±180° Y: ±90°	0.05°	50 Hz
Angular Velocity	±2000°/s	0.05°/s	50 Hz

**Table 2:** The Friedman test results of three time periods for each parameter.

Features	$a_{x}$	$a_{y}$	$a_{z}$	$w_{x}$	$w_{y}$	$w_{z}$	$g_{x}$	$g_{y}$	$g_{z}$
MAD	0.113	0.056	0.494	0.327	0.662	0.390	0.327	0.943	0.662
Kurtosis	0.838	0.120	0.113	0.838	0.113	0.193	0.113	0.230	0.014 *
Skewness	0.790	0.494	0.028 *	0.080	0.589	0.662	0.465	0.161	0.035 *
RMS	0.080	0.028 *	0.390	0.025 *	0.589	0.327	0.028 *	0.014 *	0.002 *
Variance	0.204	0.028 *	0.662	0.056	0.790	0.193	0.113	0.943	0.943
Maximum	0.056	0.465	0.193	0.028 *	0.790	0.080	0.001 *	0.080	0.002 *
Minimum	0.465	0.001 *	0.193	0.494	0.113	0.790	0.494	0.790	0.005 *
Range	0.023 *	0.465	0.193	0.059	0.790	0.193	0.001 *	0.080	0.005 *
Median	0.001 *	0.494	0.028 *	0.589	0.790	0.390	0.662	0.327	0.005 *
EC	0.193	0.028 *	0.230	0.019 *	0.790	0.291	0.028 *	0.014 *	0.000 *

* Significance level refers to *p* < 0.05 in the Friedman test, MAD refers to median absolute deviation, RMS refers to root mean square, and EC refers to energy consumption.

**Table 3:** Post hoc analysis on outcome variables with significance in the Friedman test.

Signals	Features	Baseline-30th		Baseline-60th		30th–60th	
		*p*	Adjusted *p*	*p*	Adjusted *p*	*p*	Adjusted *p*
$a_{x}$	range	0.17	0.51	0.006	0.018 *	0.17	0.51
	median	0.17	0.51	0	0 *	0.016	0.049 *
$a_{y}$	RMS	0.016	0.049 *	1	1	0.026	0.077
	variance	0.016	0.049 *	1	1	0.026	0.077
	minimum	0.303	0.91	0.01	0.03 *	0	0.001 *
	EC	0.016	0.049 *	1	1	0.026	0.077
$a_{z}$	skewness	1	1	0.016	0.049 *	0.026	0.077
	median	1	1	0.026	0.077	0.016	0.049 *
$w_{x}$	RMS	1	1	0.01	0.03 *	0.04	0.119
	maximum	1	1	0.016	0.049 *	0.026	0.077
	EC	1	1	0.006	0.018 *	0.059	0.178
$g_{x}$	RMS	0.026	0.077	0.016	0.049 *	1	1
	maximum	0.016	0.049 *	0	0 *	0.17	0.51
	range	0.016	0.049 *	0	0 *	0.17	0.51
	EC	0.026	0.077	0.016	0.049 *	1	1
$g_{y}$	RMS	0.23	0.69	0.004	0.011 *	0.086	0.259
	EC	0.23	0.69	0.004	0.011 *	0.086	0.259
$g_{z}$	kurtosis	0.23	0.69	0.004	0.011 *	0.086	0.259
	skewness	0.303	0.91	0.01	0.03 *	0.123	0.368
	RMS	0.002	0.006 *	0.002	0.006 *	1	1
	maximum	0.004	0.011 *	0.001	0.003 *	1	1
	minimum	0.006	0.018 *	0.004	0.011 *	1	1
	range	0.004	0.011 *	0.006	0.018 *	1	1
	median	0.004	0.011 *	0.006	0.018 *	1	1
	EC	0	0.001 *	0	0.001 *	1	1

* Significance level refers to *p* < 0.05 in the Wilcoxon signed-rank test, MAD refers to median absolute deviation, RMS refers to root mean square, and EC refers to energy consumption.
